# Direct dapagliflozin exposure enhances respiration and membrane hyperpolarization in isolated cardiac mitochondria

**DOI:** 10.3389/fphys.2026.1716764

**Published:** 2026-05-01

**Authors:** Itanna Isis Araújo de Souza, César Francisco Maricato da Rosa, Laís Eduardo Marinho, Marcella Borges Coutinho, Caroline Da Silva Moraes, Antonio Carlos Campos de Carvalho, José Hamilton Matheus Nascimento, Leonardo Maciel de Oliveira Pinto

**Affiliations:** 1 Instituto de Biofísica Carlos Chagas Filho, Universidade Federal do Rio de Janeiro, Rio de Janeiro, RJ, Brazil; 2 Universidade Federal do Rio de Janeiro, Duque de Caxias, RJ, Brazil; 3 Programa de Pós-Graduação Em Cardiologia, Universidade Federal do Rio de Janeiro, Rio de Janeiro, RJ, Brazil

**Keywords:** mitochondria, mitochondria electrophysiology, mitochondrial calcium handling, redox, sodium-glucose cotransporter 2 inhibitors

## Abstract

Dapagliflozin, a sodium–glucose cotransporter 2 (SGLT2) inhibitor widely used for the treatment of diabetes, has been consistently associated with cardiovascular protection, including attenuation of ischemia/reperfusion injury and reduced incidence of heart failure. However, the cellular and molecular mechanisms underlying these effects remain incompletely understood. In this context, the present study aimed to investigate whether dapagliflozin exerts direct effects on mitochondrial function and bioenergetics. Cardiac mitochondria were isolated from Wistar rats (Rattus norvegicus), and mitochondrial function was systematically evaluated by assessing oxygen consumption, ATP production, reactive oxygen species (ROS) generation, and mitochondrial membrane potential following exposure to dapagliflozin (10 nM). Dapagliflozin increased oxygen consumption in states 1–3 supported by complex I substrates and enhanced both basal and ADP-stimulated respiration in complex II, without affecting state 4 respiration, complex IV activity, or maximal uncoupled respiration. In parallel, dapagliflozin significantly reduced mitochondrial ROS production in both complexes I and II without altering ATP generation, resulting in an increased ATP/ROS ratio, indicative of improved bioenergetic efficiency. Notably, electron leakage was increased in complex I but remained unchanged in complex II, suggesting differential modulation of electron transport chain components. Furthermore, dapagliflozin induced mitochondrial membrane hyperpolarization in the presence of Ca^2+^, with or without oligomycin, and to a lesser extent in the presence of K⁺, while no significant effects were observed under Na⁺ conditions. Collectively, these findings demonstrate that dapagliflozin directly modulates mitochondrial bioenergetics and redox balance, supporting a mechanistic link between mitochondrial function and its cardioprotective effects.

## 1 Introduction

Dapagliflozin and its analogs are selective sodium-glucose cotransporter 2 (SGLT2) inhibitors, initially developed to improve glycemic control in type 2 diabetes mellitus, a highly prevalent comorbidities strongly linked to cardiovascular and renal complications (Dhillon, 2019; Belosludtseva et al., 2021). Beyond its canonical glucosuric and natriuretic effects, accumulating evidence suggested potential cardiovascular benefits, providing the rationale SGLT2 inhibitors as a therapeutic option in heart disease (Zinman et al., 2015; McMurray et al., 2019).

Experimental assays with SGLT inhibitors have consistently demonstrated cardioprotective effects against malignant cardiac conditions (Andreadou et al., 2020; Sayour et al., 2021). Preclinical studies indicate that SGLT2 inhibitors improve myocardial function following ischemia/reperfusion (I/R) injury by reducing infarct size, suppressing ventricular arrhythmias, and attenuating cardiomyocyte apoptosis (Andreadou et al., 2020; Lahnwong et al., 2020). Mechanistically, these benefits were attributed to enhanced mitochondrial bioenergetics, stimulation of mitochondrial biogenesis, stabilization of mitochondrial dynamics, and mitigation of oxidative stress (Dabravolski et al., 2022; Goedeke et al., 2024). Additional mechanisms include attenuation of ferroptosis through inhibition of the MAPK pathway, and preservation of microvascular and endothelial integrity via modulation of the XO–SERCA2–CaMKII–cofilin signaling axis (Ma et al., 2022; Chen et al., 2023). Beyond the setting of acute myocardial infarction, SGLT2 inhibitors exert beneficial effects in chronic heart failure models. For instance, SGLT2 inhibitors prevented cardiac remodeling by reducing macrophage-mediated inflammation (Wu et al., 2023). Moreover, in doxorubicin-induced heart failure models, empagliflozin improved myocardial strain, decreased cardiac fibrosis, and suppressed pro-inflammatory cytokine release, partly through mechanisms involving SIRT6-dependent pathways and oxidative-stress reduction (Sabatino et al., 2020; Ma et al., 2024).

Translation of these mechanistic insights into clinical practice has been supported by randomized controlled trials, which consistently demonstrated that SGLT2 inhibitors significantly reduce the composite endpoint of heart failure hospitalization and cardiovascular mortality in patients with heart failure with reduced ejection fraction (HFrEF), regardless of diabetic status (McMurray et al., 2019; Packer et al., 2020). Moreover, efficacy has been observed in patients with preserved or mildly reduced ejection fraction (HFpEF and HFmrEF), with improvements in symptomatic burden and attenuation of adverse cardiovascular outcomes (Anker et al., 2021). In acute decompensated heart failure, SGLT2 inhibition facilitated more rapid clinical improvement and decongestion with early clinical benefit after in-hospital initiation (Voors et al., 2022; Cox et al., 2024).

While the natriuretic, glucosuric, and hemodynamic effects of SGLT2 inhibitors undoubtedly contribute to cardiac protection (Lopaschuk and Verma, 2020), these canonical actions alone cannot fully explain the durable benefits on survival, reverse remodeling, and functional capacity observed in chronic heart failure (Lopaschuk and Verma, 2020). Given that heart failure is characterized by profound metabolic remodeling, emerging evidence suggests that mitochondrial preservation and enhancement of myocardial energetics represent central mechanisms underlying the cardioprotective actions of SGLT2 inhibitors (Dabravolski et al., 2022; Goedeke et al., 2024). Elsewhere in systemic effects, these SGLT2 inhibitors could preserve mitochondrial bioenergetics by sustaining oxidative phosphorylation and ATP production, thereby enhancing myocardial energy efficiency (Dabravolski et al., 2022). Importantly, SGLT2 inhibition stabilizes mitochondrial dynamics by restoring the balance between fission and fusion processes, via modulation of DRP1, MFN1/2, and OPA1, thus preventing excessive mitochondrial fragmentation (Koizumi et al., 2023). In parallel, the promotion of mitophagy contributes to the clearance of damaged organelles and maintains mitochondrial quality control (Yang et al., 2024). Another crucial mechanism involves the attenuation of mitochondrial ROS production and reinforcement of antioxidant defenses, which collectively limit oxidative stress-induced injury (Dabravolski et al., 2022; Koizumi et al., 2023). Furthermore, SGLT2 inhibitors optimize ionic homeostasis, lowering cytosolic Na^+^ and Ca²^+^ while preserving mitochondrial Ca²^+^ handling (Baartscheer et al., 2017; Uthman et al., 2018b), thereby preventing Ca²^+^ overload and ensuring proper coupling between energy demand and supply (Lopaschuk and Verma, 2020).

Collectively, these pleiotropic mitochondrial effects converge to attenuate cardiomyocyte apoptosis, limit maladaptive remodeling, and preserve myocardial resilience across ischemic and non-ischemic models of heart failure (Dabravolski et al., 2022; Wu et al., 2023). Nevertheless, despite these consistent experimental findings, the precise mechanisms by which SGLT2 inhibitors act on mitochondria remain incompletely understood. Therefore, it is unclear whether these agents could interact directly with mitochondrial structures, or whether their actions are mediated indirectly through upstream signaling pathways. This unresolved question underscores the need for further mechanistic studies to define whether SGLT2 inhibitors exert a direct mitochondrial effect or operate via systemic and cytosolic signaling cascades (Lopaschuk and Verma, 2020; Dabravolski et al., 2022).

Thus, the present study intent to investigate the effects of dapagliflozin on mitochondria isolated from cardiac tissue, to explore the potential direct actions of the drug at the organelle level.

## 2 Methods

### 2.1 Materials

All chemicals (analytical grade) were obtained from Sigma-Aldrich (USA), unless otherwise specified. All solutions were freshly prepared and filtered (1.2 μm, Millipore).

### 2.2 Animals and ethical approval

This study complied with the *Guide for the Care and Use of Laboratory Animals* published by the US National Institutes of Health (8th edition, 2011). The experimental protocols were approved by the Institutional Animal Care and Use Committee (protocol 023/25, approved in 2022). Animals were housed under controlled conditions with a 12:12-h light–dark cycle and a stable temperature of 23–24 °C, with free access to food and water. A total of 6 male Wistar rats per group (4 weeks old) were used. Mitochondria isolated from the control group were simultaneously used for exposure to Dapagliflozin at a concentration of 10 nM/mL.

### 2.3 Mitochondria isolation

Mitochondria were isolated following previously established protocols (24 – Gedik et al., 2017). Rats were anesthetized by intraperitoneal injection of sodium Thiopental (400 mg/kg; Cristália, Rio de Janeiro, Brazil) and euthanized by decapitation. A thoracotomy was performed to carefully extract the heart, which was immediately placed in ice-cold isolation buffer (250 mmol/L sucrose, 10 mmol/L HEPES, 1 mmol/L ethylene glycol tetraacetic acid [EGTA]; pH 7.4, adjusted with TRIS) maintained at 4 °C to remove excess blood. To optimize the procedure, 0.5% (w/v) bovine serum albumin (BSA) was added to the buffer. Adipose tissue and atrium were meticulously removed with scissors, and the heart was minced into ~1–2 mm pieces, ensuring complete fat removal. The tissue was homogenized using an Ultra-Turrax homogenizer (two 10-s cycles at 6500 rpm, on ice). The homogenate was further processed with a Potter–Elvehjem homogenizer with a Teflon pestle, assisted by proteinase type XXIV (8 IU/mg tissue weight) for 2 minutes. The homogenate was centrifuged at 700 × g for 10 min at 4 °C, and the supernatant was collected and centrifuged again at 12300 × g for 10 min. The pellet was resuspended in ice-cold isolation buffer without BSA and centrifuged once more at 10000 × g for 10 min at 4 °C. This washing step was repeated to ensure purity. The final pellet was resuspended in isolation buffer. Protein concentration was determined by the Lowry assay (Bio-Rad, Hercules, CA, USA) using bovine serum albumin (Thermo Scientific, Waltham, MA, USA) as the standard.

### 2.4 Mitochondrial oxygen consumption protocol

Mitochondrial respiration for complexes I (states 1, 2, and 3) complex IV, and maximal uncoupling oxygen uptake, was evaluated using a two-chamber respirometer. Measurements were performed with a Clark-type oxygen electrode (Strathkelvin, Glasgow, UK) at 37 °C under magnetic stirring in an incubation buffer. The buffer composition included 125 mmol/L KCl, 10 mmol/L MOPS, 2 mmol/L MgCl_2_, 5 mmol/L KH_2_PO_4_, and 0.2 mmol/L EGTA, pH 7.2, adjusted with TRIS, with substrates such as glutamate (5 mmol/L) and malate (5 mmol/L) for complex I or succinate (5 mmol/L) for complex II. The oxygen electrode was calibrated using a solubility coefficient of 217 nmol O_2_/mL at 37 °C. To measure complex I respiration, mitochondria (containing 100 µg of mitochondrial protein) were added to 1 mL of the incubation buffer. After 2 minutes of equilibration, ADP (1 mmol/L) was introduced, and ADP-stimulated respiration was recorded over a 2-minute interval. For complex II respiration, the complex I inhibitor rotenone (1 µmol/L) was added. Subsequent measurements of complex IV respiration and maximal uncoupled oxygen uptake were performed directly in the respiration chamber, Complex IV respiration was stimulated by adding N,N,N’,N’-tetramethyl-p-phenylenediamine (TMPD, 300 µmol/L) and ascorbate (3 mmol/L), which serve as electron donors to cytochrome oxidase by reducing cytochrome c. Maximal uncoupled oxygen consumption was determined in the presence of 30 nmol/L carbonyl cyanide-p-trifluoromethoxyphenylhydrazone (FCCP). For each experimental condition, the experimental protocol was repeated until the ADP addition step, when aliquots of the mitochondrial incubation buffer were collected for the assessment of ATP production and mitochondrial ROS levels (24 – Gedik et al., 2017).

### 2.5 Mitochondrial ATP production

**Figure 1:**
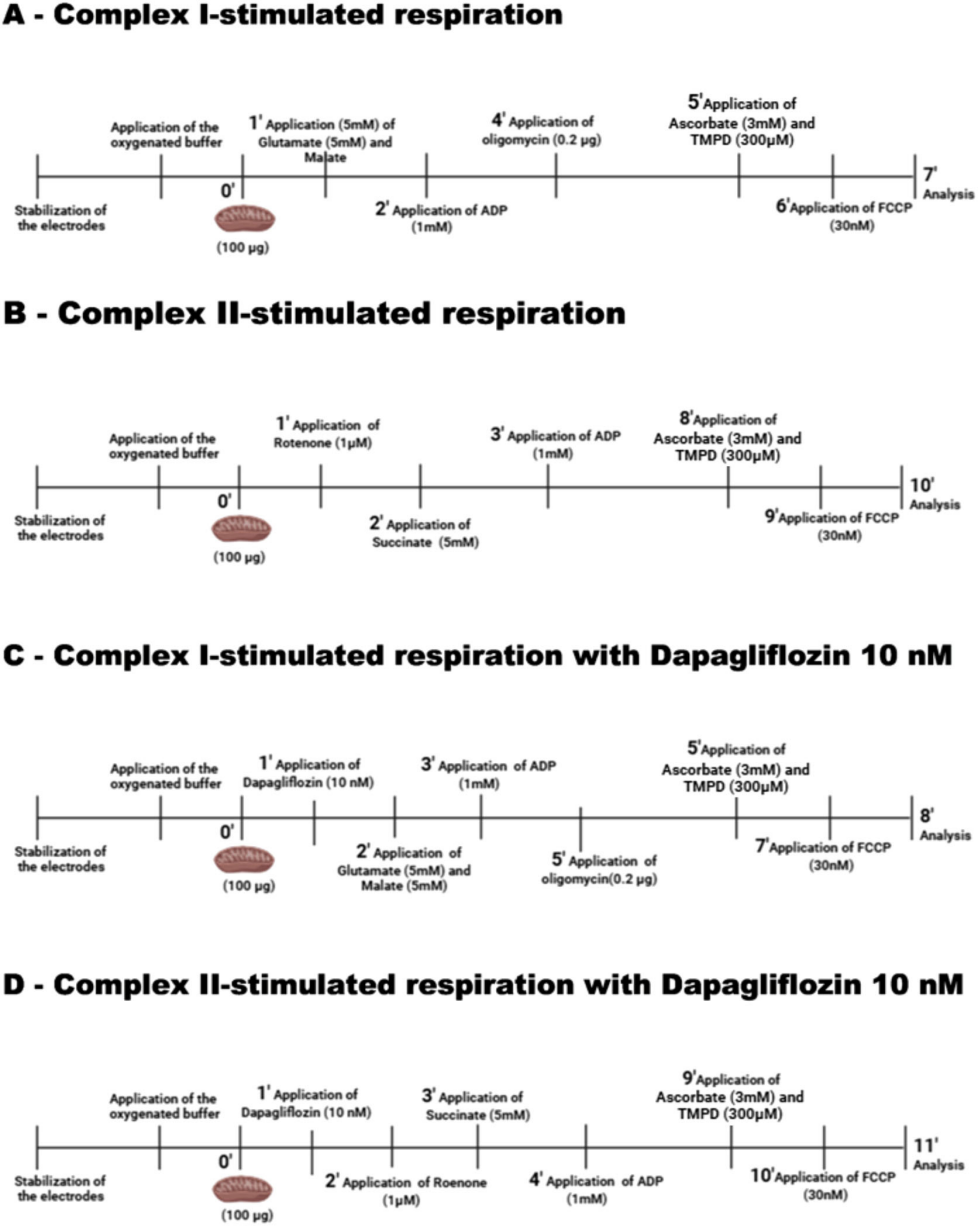
Experimental protocols for mitochondrial oxygen consumption. Oxygen consumption was measured stepwise in isolated mitochondria under four different conditions: **(A)** Complex I–stimulated respiration; **(B)** Complex II–stimulated respiration; **(C)** Complex I–stimulated respiration in the presence of dapagliflozin (10 nM); and **(D)** Complex II–stimulated respiration in the presence of dapagliflozin (10 nM). In all protocols, measurements included electrode stabilization, addition of mitochondria (100 µg), and sequential substrate/inhibitor additions as indicated in each panel. For Complex I–driven respiration **(A, C)**, glutamate (5 mM) and malate (5 mM) were added, followed by ADP (1 mM), oligomycin (0.2 µg), ascorbate (3 mM) plus TMPD (300 µM), and FCCP (30 nM). For Complex II–driven respiration **(B, D)**, rotenone (1 µM) was first added to inhibit Complex I, followed by succinate (5 mM), ADP (1 mM), ascorbate (3 mM) plus TMPD (300 µM), and FCCP (30 nM). Where indicated **(C, D)**, dapagliflozin (10 nM) was added prior to substrate injection. Time points and sequence of additions are shown schematically in each panel.

ATP formation was assessed in parallel and independent experiments, distinct from the mitochondrial respiration assays shown in **Figure 1**. Isolated mitochondria were incubated in the presence of energetic substrates for Complex I or Complex II respiration, together with ADP (1 mM). After a fixed incubation period of 3 minutes, samples were rapidly collected for ATP quantification. ATP concentration was determined using a bioluminescence-based assay (ATP Bioluminescence Assay Kit, Sigma-Aldrich, St. Louis, MO, USA). ATP levels were quantified by comparison with ATP standard curves using a 96-well white plate in a spectrofluorometer (SpectraMax^®^ M3, Molecular Devices, San Jose, CA, USA) with emission at 560 nm (Gedik et al., 2017; Mesquita et al., 2022; de Souza et al., 2024).

### 2.6 Mitochondrial ROS concentration

Reactive oxygen species (ROS) levels were measured using the Amplex Red Hydrogen Peroxide Assay Kit (Life Technologies, Carlsbad, CA, USA). Amplex Red reacts with hydrogen peroxide in a 1:1 stoichiometry in the presence of horseradish peroxidase (HRP), forming highly fluorescent resorufin with 95% efficiency. The incubation buffer was collected from the respiration chamber and immediately supplemented with 50 μmol/L Amplex UltraRed and 2 U/mL Pierce™ HRP (Thermo Fisher, Catalog No. 31491, Waltham, MA, USA) (Gedik et al., 2017; Mesquita et al., 2022; de Souza et al., 2024).

### 2.7 Mitochondrial transmembrane potential

The mitochondrial transmembrane potential (Δψm) was evaluated using a Cary Eclipse Fluorescence Spectrometer (Agilent, CA, United States) as previously described (de Souza et al., 2024). Isolated mitochondria (100 μg/mL) were suspended in respiration medium with composition included 125 mmol/L KCl, 10 mmol/L MOPS, 2 mmol/L MgCl_2_, 5 mmol/L KH_2_PO_4_, pH 7.2, adjusted with TRIS (final volume 2 mL) containing glutamate (5 mmol/L) and malate (5 mmol/L) as respiratory substrates. The experiments were performed at 37 °C under continuous magnetic stirring to maintain homogeneous suspension and optimal oxygen diffusion. The fluorescent probe tetramethylrhodamine methyl ester (TMRM, 400 nmol) was used to monitor changes in mitochondrial membrane potential. Measurements were performed in the presence of either ADP (1 mmol/L). Fluorescence signals were continuously recorded in arbitrary units (AU). To investigate the effects of ionic stress on mitochondrial polarization, mitochondria were exposed to progressively increasing concentrations of calcium, or sodium, or potassium. Mitochondria were exposed to sequential pulses of KCl, or CaCl_2_, or NaCl. Each salt was added in the mitochondrial suspension in increments of 2.5 µM at 1-minute intervals, generating stepwise increases in the ionic concentration within the cuvette. Mitochondrial membrane potential was continuously monitored for 10 minutes, allowing the evaluation of the dynamic response of Δψm to the progressive ionic challenge. In additional experiments, freshly isolated mitochondria were incubated with Dapagliflozin (10 nM) to assess potential modulatory effects on mitochondrial Δψm. The mitochondrial suspension was placed in a quartz cuvette and fluorescence was recorded continuously over time. Experimental conditions were distributed in separate cuvettes: (1) mitochondria incubated with respiratory substrates and vehicle; (2) mitochondria incubated with respiratory substrates and Dapagliflozin; (3) mitochondria exposed to FCCP, used as a positive control to induce maximal mitochondrial depolarization; and (3) mitochondria treated with cyclosporine A (CSA) to evaluate the involvement of the mitochondrial permeability transition pore. The mitochondrial membrane potential was quantified by comparing the baseline fluorescence at the start of the experiment with the fluorescence values recorded at 1-minute intervals over a 10-minute period, allowing the determination of time-dependent changes in Δψm under each experimental condition (Gedik et al., 2017; Mesquita et al., 2022; de Souza et al., 2024).

### 2.8 Electron leakage and ATP/ROS production ratio

Electron leakage corresponds to the escape of electrons from the respiratory chain to form superoxide (O2−). Other ROS, such as hydroperoxyl radical (HO2) and hydrogen peroxide (H2O2), may arise spontaneously (e.g., in a pH-dependent manner) or through enzymatic conversion (e.g., by superoxide dismutase). The initial leakage site is generally attributed to semiquinone radicals (QH^•^) or reduced flavins (FMN, FAD). Electron leakage was calculated as the ratio between H2O2 formation and mitochondrial O2 consumption. Measurements were performed using complex I under State 3 respiration. The ATP/ROS ratio was determined to assess the balance between oxygen consumption leading to ATP synthesis and ROS generation (Gedik et al., 2017; Mesquita et al., 2022; de Souza et al., 2024).

### 2.9 Statistical analysis

Data were presented as the mean ± standard deviation (SD). For graphic and statistical analysis, the software GraphPad Prism 8.4.3 (San Diego, CA, USA) was used. The data distribution was considered normal by the Shapiro–Wilk test. The significant differences in mitochondrial oxygen consumption and the functions were evaluated by the parametric Student *t*-test. *p* < 0.05 was considered statistically significant. The data was analyzed by Two-Way ANOVA, with Bonferroni post-test when appropriated. This section is not mandatory but can be added to the manuscript if the discussion is unusually long or complex.

## 3 Results

### 3.1 Mitochondrial oxygen consumption

**Figure 2:**
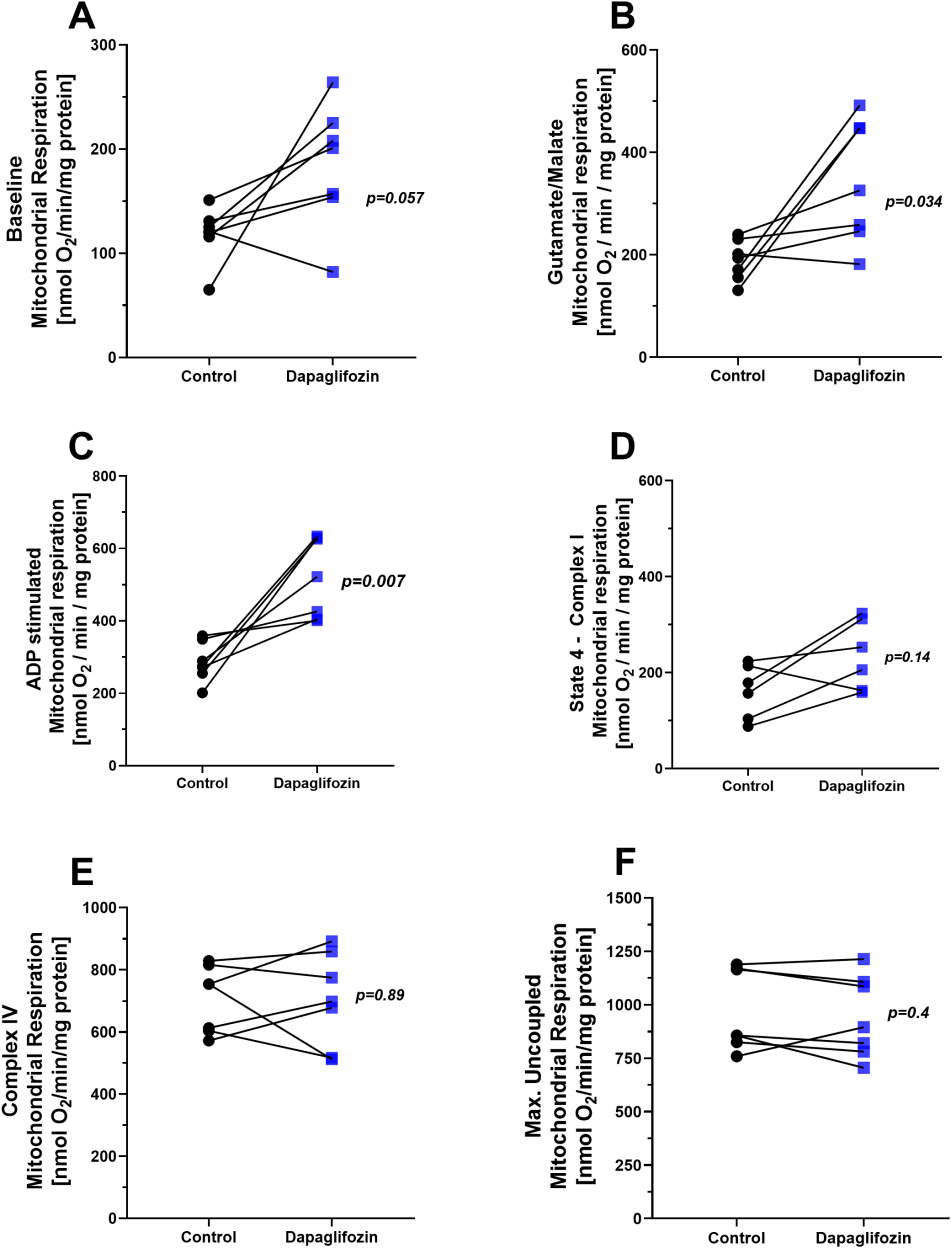
Mitochondrial respiration in the presence of dapagliflozin (10 nM). **(A)** Basal respiration (state 1, complex I), **(B)** glutamate/malate (5mM/5mM)-stimulated respiration (state 2, complex I), **(C)** ADP(1mM)-stimulated respiration (state 3, complex I; phosphorylative state), **(D)** state 4 respiration in complex I induced by oligomycin (0.2µg/ml), **(E)** complex IV respiration stimulated by TMPD (300µM) and ascorbate (3 mM), and **(F)** maximal uncoupled respiration induced by FCCP (30 nM) in isolated mitochondria from rat hearts. Each experiment represents one animal. Black circles indicate the control condition, whereas blue squares represent the Dapagliflozin-treated condition. Both conditions were assessed in parallel using the same mitochondrial preparation (paired design). Statistical analysis was conducted using the paired Student’s t-test.

The Oxygen consumption of isolated mitochondria was analyzed following exposure to Dapagliflozin at a concentration of 10 nM (**Figure 2**). In state 1 of complex I, mitochondrial Oxygen consumption tended to be higher in the Dapagliflozin group compared to the control group (**Figure 2A**), however without statistical significance. In state 2 of complex I, where oxygen consumption was stimulated by glutamate and malate, mitochondria exposed to Dapagliflozin showed an increase in oxygen consumption relative to the control group (**Figure 2B**), *p=0.034*. Similarly, in state 3 of complex I, ADP-stimulated respiration was higher in the Dapagliflozin group compared to the control group (**Figure 2C**), *p=0.007*. In contrast, in state 4 of Complex I (oligomycin induced leak respiration), no differences were observed between groups (**Figure 2D**). No statistically significant differences were observed in mitochondrial respiration through complex IV (**Figure 2E**) or in uncoupled maximal oxygen consumption (**Figure 2F**), suggesting similar mitochondrial loading controls and viability, respectively, across groups.

**Figure 3:**
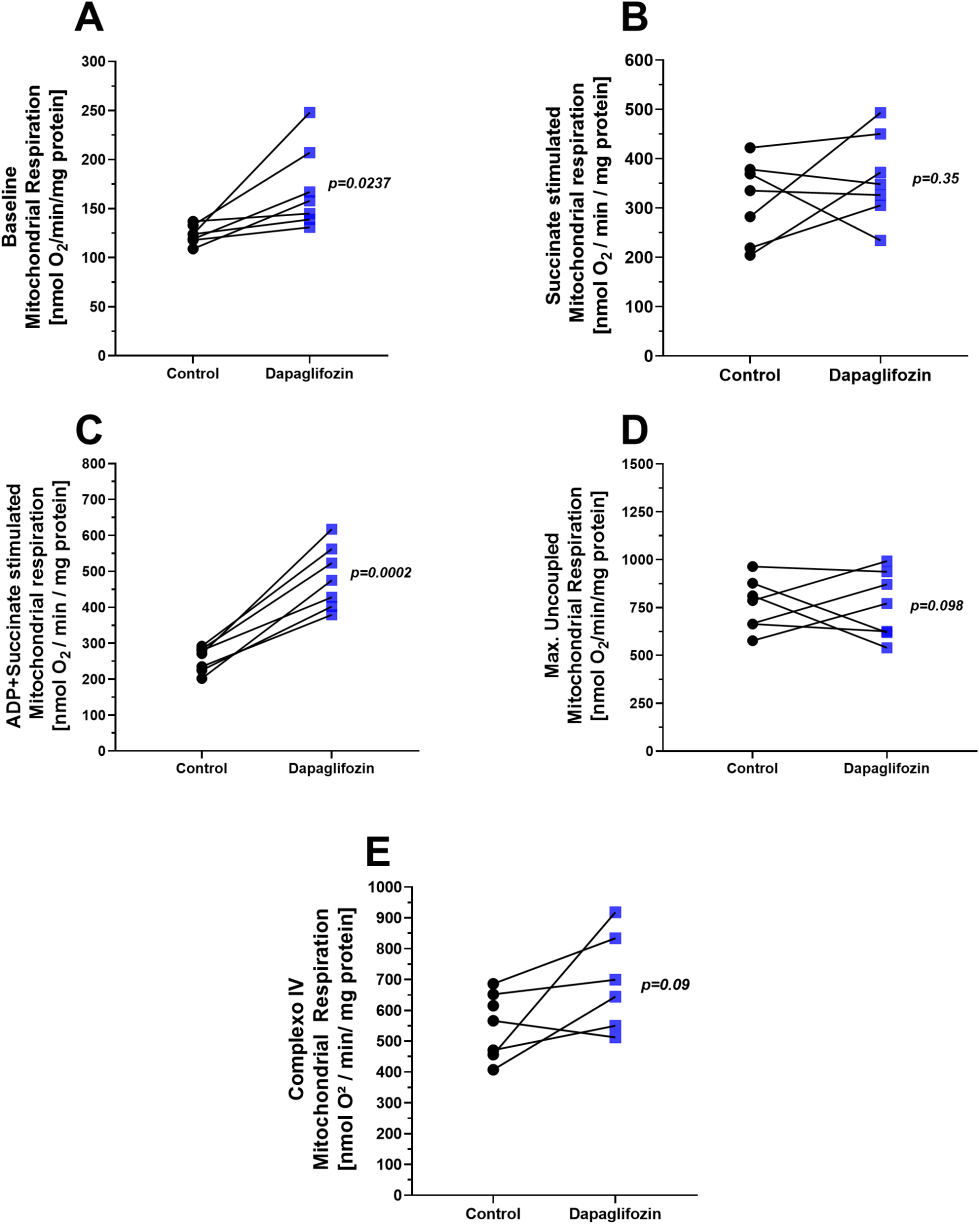
Mitochondrial respiration in the presence of dapagliflozin (10 nM). **(A)** Basal respiration (state 1), measured with complex I inhibition by rotenone (1 µM), **(B)** succinate (5 mM)-stimulated respiration (state 2, complex II), **(C)** ADP (1 mM)-stimulated respiration (state 3, complex II; phosphorylative state), **(D)** maximal uncoupled respiration induced by FCCP (30 nM), and **(E)** complex IV respiration stimulated by TMPD (300 µM) and ascorbate (3 mM) in isolated mitochondria from rat hearts. Each experiment represents one animal. Black circles indicate the control condition, whereas blue squares represent the Dapagliflozin-treated condition. Both conditions were assessed in parallel using the same mitochondrial preparation (paired design). Statistical analysis was conducted using the paired Student’s t-test.

Mitochondrial respiration stimulated by complex II was assessed following exposure to 10 nM Dapagliflozin (**Figure 3**). At state 1, basal respiration was elevated in the Dapagliflozin group compared to the control (**Figure 3A**), *p=0.0237*. When oxygen consumption was subsequently stimulated with Succinate in state 2, oxygen consumption increased similarly in both groups, with no significant differences observed (**Figure 3B**). In state 3, ADP-stimulated respiration in the presence of succinate was significantly higher in the Dapagliflozin exposed mitochondria relative to the control group (**Figure 3C**), *p=0.0002*. Both, maximal uncoupled respiration (**Figure 3D**) and complex IV respiration (**Figure 3E**) remained unchanged between the groups, suggesting similar mitochondrial loading controls and viability, respectively, across groups. Representative traces can be found in **Figure 1** of the Supplementary Material.

### 3.2 Mitochondrial ROS production, ATP production, mitochondrial electron leakage and ATP/ROS ratio

**Figure 4:**
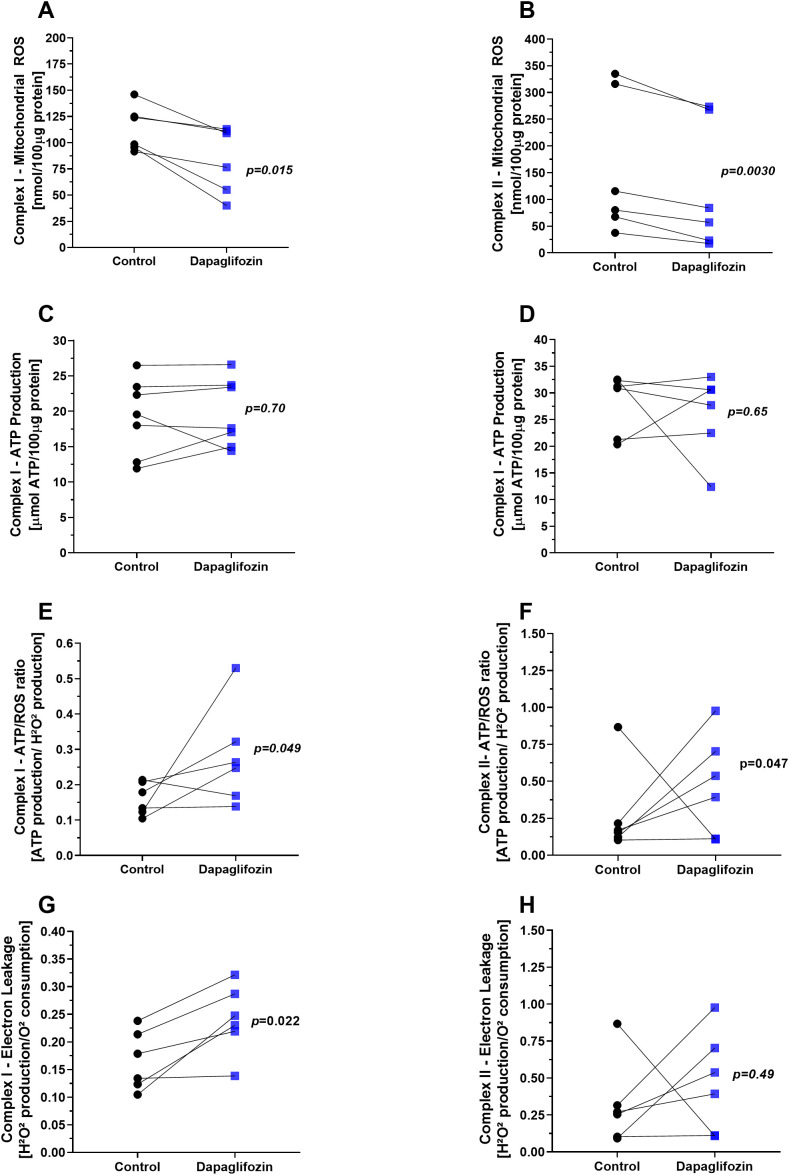
Mitochondrial products and functional parameters in the presence of dapagliflozin (10 nM). **(A)** Reactive oxygen species (ROS) production at complex I, **(B)** ROS production at complex II, **(C)** adenosine triphosphate (ATP) production at complex I, **(D)** ATP production at complex II, **(E)** ATP/ROS ratio at complex I, **(F)** ATP/ROS ratio at complex II, and **(G)** electron leak at complex **(I)** Each symbol represents one animal. Each experiment represents one animal. Black circles indicate the control condition, whereas blue squares represent the Dapagliflozin-treated condition. Both conditions were assessed in parallel using the same mitochondrial preparation (paired design). Statistical analysis was conducted using the paired Student’s t-test.

Mitochondrial reactive oxygen species (ROS) production stimulated by complex I showed that exposure to Dapagliflozin significantly reduced ROS levels compared to the control group (**Figure 4A**), *P=0.015*. Similarly, ROS production by complex II was also significantly decreased following incubation with Dapagliflozin (**Figure 4B**), *p=0.003*. ATP production by complex I did not show significant changes with Dapagliflozin treatment (**Figure 4C**). Likewise, ATP production by complex II was not significantly altered by Dapagliflozin (**Figure 4D**). The ATP/ROS ratio of complex I was significantly increased in the presence of Dapagliflozin compared to the control group (**Figure 4E**), *p=0.049*, indicating that ROS production by complex I was proportionally lower relative to ATP synthesis. Similarly, the ATP/ROS ratio of complex II was also significantly elevated with Dapagliflozin relative to the control group (**Figure 4F**), *p=0.047*, indicating that ROS production by complex II was proportionally lower relative to ATP synthesis. Analysis of electron leak from complex I revealed a significant increase after exposure to Dapagliflozin compared to the control group (**Figure 4G**), *p=0.022*. On the other hand, electron leakage from complex II showed no significant differences between the groups (**Figure 4H**).

### 3.3 Mitochondrial transmembrane potential (Δψm)

**Figure 5:**
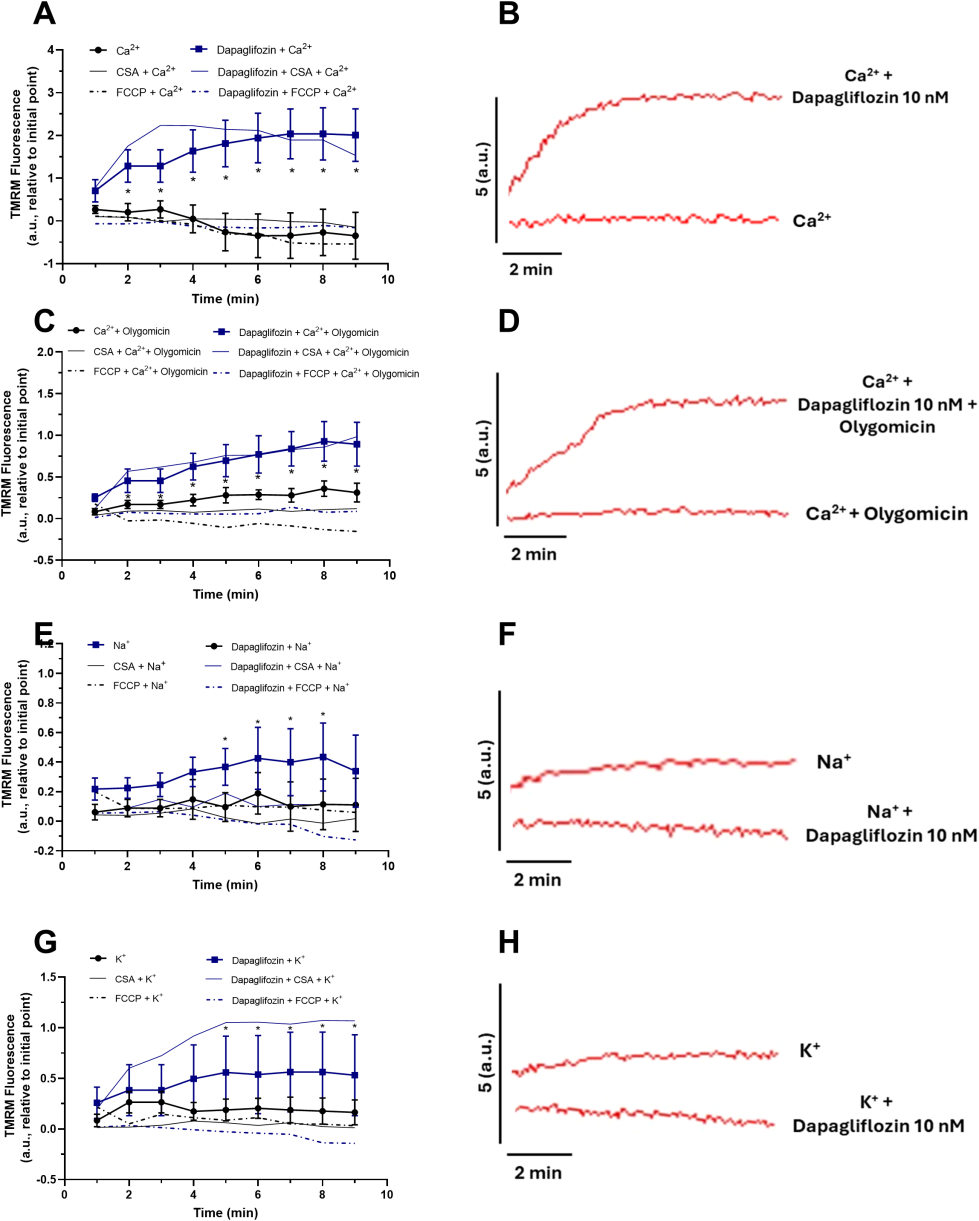
Mitochondrial transmembrane potential (Δψm) in isolated rat heart mitochondria exposed to dapagliflozin (10 nM). **(A)** Quantification of Δψm in the presence of Ca²^+^ (2.5 mM). **(B)** Representative fluorescence traces showing Δψm in response to sequential Ca²^+^ pulses applied every minute, in the absence (control) or presence of Dapagliflozin. **(C)** Quantification of Δψm in the presence of Ca²^+^ (2.5 mM) and oligomycin (0.2 µg/mL). **(D)** Representative traces showing Δψm with Ca²^+^ + oligomycin in control and Dapagliflozin-treated groups. **(E)** Quantification of Δψm in the presence of Na^+^ (2.5 mM). **(F)** Representative fluorescence traces showing Δψm after Na^+^ pulses applied every minute, in the absence or presence of Dapagliflozin. **(G)** Quantification of Δψm in the presence of K^+^ (2.5 mM). **(H)** Representative traces showing Δψm after K^+^ pulses applied every minute, in the absence or presence of Dapagliflozin. Fluorescence was recorded using TMRM (400 nM) in mitochondrial suspensions (100 μg protein/mL) energized with glutamate (5 mM) and malate (5 mM), in the presence of ADP (1 mM) or oligomycin (0.2 µG/mL), at 37 °C under constant stirring. Data are expressed relative to baseline values (minute 0). Each symbol represents one animal. All conditions were assessed in parallel using the same mitochondrial preparation (paired design). Statistical analysis was conducted using the Two-Way ANOVA, with Bonferroni post-test. The Data were expressed by Mean± Standard deviation. n=6 for each group.

The addition of Ca²^+^ in the presence of Dapagliflozin induced a progressive increase in fluorescence compared with Ca²^+^ alone, indicating mitochondrial membrane hyperpolarization (**Figure 5A**). Representative traces illustrate that Dapagliflozin combined with Ca²^+^ markedly increased fluorescence relative to the control (**Figure 5B**). When mitochondria were exposed to Ca²^+^, a mitochondrial membrane depolarization was observed even in the presence of oligomycin, which typically maintains the membrane potential in a more negative (hyperpolarized) state. In contrast, this depolarization seems to be prevented in the presence of Dapagliflozin (**Figure 5C**). Representative traces show that fluorescence remained elevated (polarized) with Dapagliflozin despite Ca²^+^ exposition (**Figure 5D**). In contrast, exposure to Dapagliflozin in the presence of Na^+^ resulted in a less pronounced response, with mitochondrial fluorescence showing no significant difference compared with the control (**Figure 5E**). Representative traces corroborate this finding, showing similar fluorescence dynamics between groups (**Figure 5F**). Finally, when mitochondria were exposed to Dapagliflozin in the presence of K^+^, a gradual increase in fluorescence was observed, although the effect was less pronounced than with Ca²^+^ (**Figure 5G**). Representative traces show a modest increase in fluorescence in the Dapagliflozin group compared with the control (**Figure 5H**).

## 4 Discussion

To our knowledge, this is the first study to demonstrate that dapagliflozin directly modulates functional parameters of isolated cardiac mitochondria. Previous evidence for mitochondrial effects of SGLT2 inhibitors has been largely indirect — derived from cellular and whole-animal studies showing improved mitochondrial function, reduced oxidative stress and shifts in substrate utilization — but direct evidence in isolated mitochondria has been lacking (Maejima, 2020; Yurista et al., 2020).Our findings show that dapagliflozin increased oxygen consumption in respiratory states stimulated by energetic substrates, suggesting that the drug favors electron flux through the respiratory chain both under phosphorylating and non-phosphorylating conditions (Belosludtseva et al., 2021; Gao et al., 2025). One possible explanation is that dapagliflozin improves electron transfer efficiency within the respiratory chain, potentially by influencing the functional interaction of complexes I and II or by facilitating electron flow through the ubiquinone pool (Murphy, 2009; Wikström and Hummer, 2012; Zhao et al., 2019). Previous studies have shown that SGLT2 inhibitors modulate mitochondrial dynamics (fission/fusion) and preserve cardiac mitochondrial integrity, for example by regulating fusion/fission proteins (Liesa et al., 2009; Maejima, 2020). Such structural effects may help maintain mitochondrial organization in a way that supports more efficient electron transport. Since these experiments were performed using isolated mitochondria, changes in mitochondrial content cannot occur. Therefore, the observation that maximal uncoupled respiration remained unchanged indicates that dapagliflozin modulates the functional state or efficiency of the existing mitochondria rather than inducing mitochondrial biogenesis (Jastroch et al., 2010; Afsar et al., 2021).

The reduction in mitochondrial reactive oxygen species (ROS) despite higher oxygen consumption represents one of the most relevant results. This finding suggests that dapagliflozin may improve the efficiency of mitochondrial redox processes, decreasing electron stalling at redox centers predisposed to ROS generation (Murphy, 2009; Hernansanz-Agustín and Enríquez, 2021). Such effects have been hypothesized in studies showing that SGLT2 inhibitors modulate mitochondrial membrane potential and enhance endogenous antioxidant buffering (Koizumi et al., 2023; Preda et al., 2024). Furthermore, findings in tubular cells revealed that dapagliflozin reduces both mitochondrial and cytosolic ROS production while altering Ca²^+^ dynamics, reinforcing the plausibility of intramitochondrial effects without excluding antioxidant system involvement (Durak et al., 2018; Lee et al., 2019). The increased ATP/ROS ratio in dapagliflozin-exposed mitochondria suggests proportionally lower oxidative “waste” per unit of ATP generated — an indicator of improved bioenergetic efficiency (Yurista et al., 2020; Choi et al., 2023a). However, an apparent paradox was observed with the increase in proton leak at complex I. One possible explanation is that increased electron flux allows electrons to pass more rapidly through the respiratory chain, reducing the possibility of electron accumulation and ROS generation (Murphy, 2009; Jastroch et al., 2010; Nanayakkara et al., 2019). Alternatively, dapagliflozin may facilitate proton dissipation mechanisms that produce less ROS or modify redox reactions within the respiratory chain in a way that reduces their interaction with oxygen (Murphy, 2009).

The observed hyperpolarization of the mitochondrial membrane potential (Δψm) in response to Ca²^+^ suggests that dapagliflozin-treated mitochondria sustain a stronger electrochemical gradient under calcium load. This effect may reflect increased proton pumping activity by the respiratory chain or a reduction in inner mitochondrial membrane permeability to H^+^ and Ca²^+^ (Raffaello et al., 2012; Szabo and Zoratti, 2014). Such hyperpolarization fits well with models in which SGLT2 inhibitors are proposed to preserve mitochondrial integrity and maintain a more stable Δψm, thereby favoring efficient coupling (Maejima, 2020; Pham et al., 2024). Improved mitochondrial regulation of Ca²^+^ entry (e.g., through tighter control of the mitochondrial calcium uniporter, MCU, or enhanced buffering capacity) could help better match energy demand with ATP production (De Stefani et al., 2015; Hernansanz-Agustín and Enríquez, 2021). The relatively modest mitochondrial response in the presence of Na^+^ suggests that many sodium-related effects associated with SGLT2 inhibition depend on cytosolic Na^+^ regulation and ion exchange systems within intact cells (e.g., NHE, NCX) (Uthman et al., 2018a; Gao et al., 2025). Indeed, recent hypotheses propose that SGLT2 inhibitors may exert off-target effects on NHE1 in cardiomyocytes, thereby modulating intracellular Na^+^/Ca²^+^ balance and indirectly benefiting mitochondria by reducing Na^+^ overload and limiting reverse Ca²^+^ entry (Uthman et al., 2018b; Zuurbier et al., 2021). The weak Na^+^ response in isolated mitochondria therefore supports the notion that sodium-mediated effects are largely secondary, not the result of direct organellar actions (Yurista et al., 2020).

The changes in mitochondrial membrane potential (Δψm) observed in this study may be related to the activity of mitochondrial ion transporters that regulate Ca²^+^, Na^+^, and K^+^ fluxes across the inner membrane (Murphy, 2009; Szabo and Zoratti, 2014). Rapid mitochondrial Ca²^+^ uptake through the mitochondrial calcium uniporter (MCU) is driven by Δψm and increases matrix [Ca²^+^], which stimulates dehydrogenases and enhances proton pumping — a mechanism that can transiently hyperpolarize or sustain the proton motive force under substrate-rich conditions (Raffaello et al., 2012; De Stefani et al., 2015). Conversely, mitochondrial Ca²^+^ extrusion via the Na^+^/Ca²^+^ exchanger (NCLX) is electrogenic and regulated by both Δψm and cytosolic Na^+^; shifts in NCLX flux therefore critically determine whether Ca²^+^ loading leads to sustained hyperpolarization or depolarization (Samanta et al., 2018; Cabral-Costa et al., 2023). LETM1-mediated Ca²^+^/H^+^ (and possibly K^+^/H^+^) exchange provides an alternative, proton-coupled mechanism for Ca²^+^ handling that alters matrix pH and the proton gradient, thereby influencing Δψm and coupling efficiency (Szabo and Zoratti, 2014). Thus, the hyperpolarization observed in dapagliflozin-treated mitochondria during Ca²^+^ exposure may reflect mechanisms by which dapagliflozin limits mitochondrial Ca²^+^ entry or attenuates Ca²^+^-induced depolarizing effects. This could involve reduced MCU-mediated Ca²^+^ uptake, modulation of NCLX/LETM1 fluxes that prevent Ca²^+^ overload, and/or decreased K^+^ conductance, thereby preserving Δψm without triggering mitochondrial permeability transition (MPTP) (Murphy, 2009; Raffaello et al., 2012).

A fundamental aspect of our experimental design is the use of isolated mitochondria, which allows the evaluation of mitochondrial bioenergetics in the absence of cytosolic signaling. Under these conditions, the observed enhancement in ADP-stimulated respiration and the modulation of mitochondrial membrane potential (Δψm) are unlikely to arise from secondary cytosolic or systemic mechanisms. Growing evidence indicates that SGLT2 inhibitors exert SGLT2-independent cardiac actions, including modulation of the mitochondrial function in cardiomyocytes. In line with this concept, recent studies report improved cardiac mitochondrial ATP production following treatment with SGLT2 inhibitors, suggesting that these agents may influence mitochondrial bioenergetics at the organelle level (Choi et al., 2023b). Moreover, functional studies demonstrate that SGLT2 inhibitors can interact with the mitochondrial respiratory chain, particularly by modulating Complex I-driven respiration, independently of SGLT2 expression (Hawley et al., 2016). Although direct intramitochondrial accumulation of Dapagliflozin has not yet been conclusively demonstrated, the convergence of these findings supports the plausibility of a direct mitochondrial interaction. Accordingly, our results obtained in isolated cardiac mitochondria suggest that dapagliflozin modulates mitochondrial bioenergetic independently of whole cell influences.

Taken together, these findings support the concept that dapagliflozin exerts pleiotropic effects on mitochondrial function, modulating multiple aspects of mitochondrial physiology, including respiration, coupling efficiency, redox balance, and ion handling. Evidence from cellular models further supports the possibility of direct mitochondrial actions. For example, studies with empagliflozin (a structurally related SGLT2 inhibitor of the same C-aryl glucoside class as dapagliflozin), suggest that this compound can penetrate mitochondria and increase intra-mitochondrial ATP levels, supporting a potential direct interaction with the organelle (Choi et al., 2023a). However, other studies propose that some mitochondrial effects of SGLT2 inhibitors may arise indirectly through metabolic signaling pathways, such as AMPK, SIRT1, or nutrient-sensing–dependent mechanisms that regulate mitochondrial dynamics and ion homeostasis (Pham et al., 2024; Gao et al., 2025). Therefore, although this study demonstrates that dapagliflozin alters the functional state of isolated mitochondria, it remains unclear whether all mitochondrial effects from dapagliflozin result from a direct interaction with mitochondrial components or from mechanisms previously established in intact cellular systems (Uthman et al., 2018a; Yurista et al., 2020).

## Data Availability

The raw data supporting the conclusions of this article will be made available by the authors, without undue reservation.
