# Supplementary material for: Direct dapagliflozin exposure enhances respiration and membrane hyperpolarization in isolated cardiac mitochondria
Source: Front Physiol. 2026 May 1;17:1716764. doi: 10.3389/fphys.2026.1716764 (PMC13175820; doi:10.3389/fphys.2026.1716764)
Supplement: Supplementary file 1 [file SupplementaryFile1.docx]

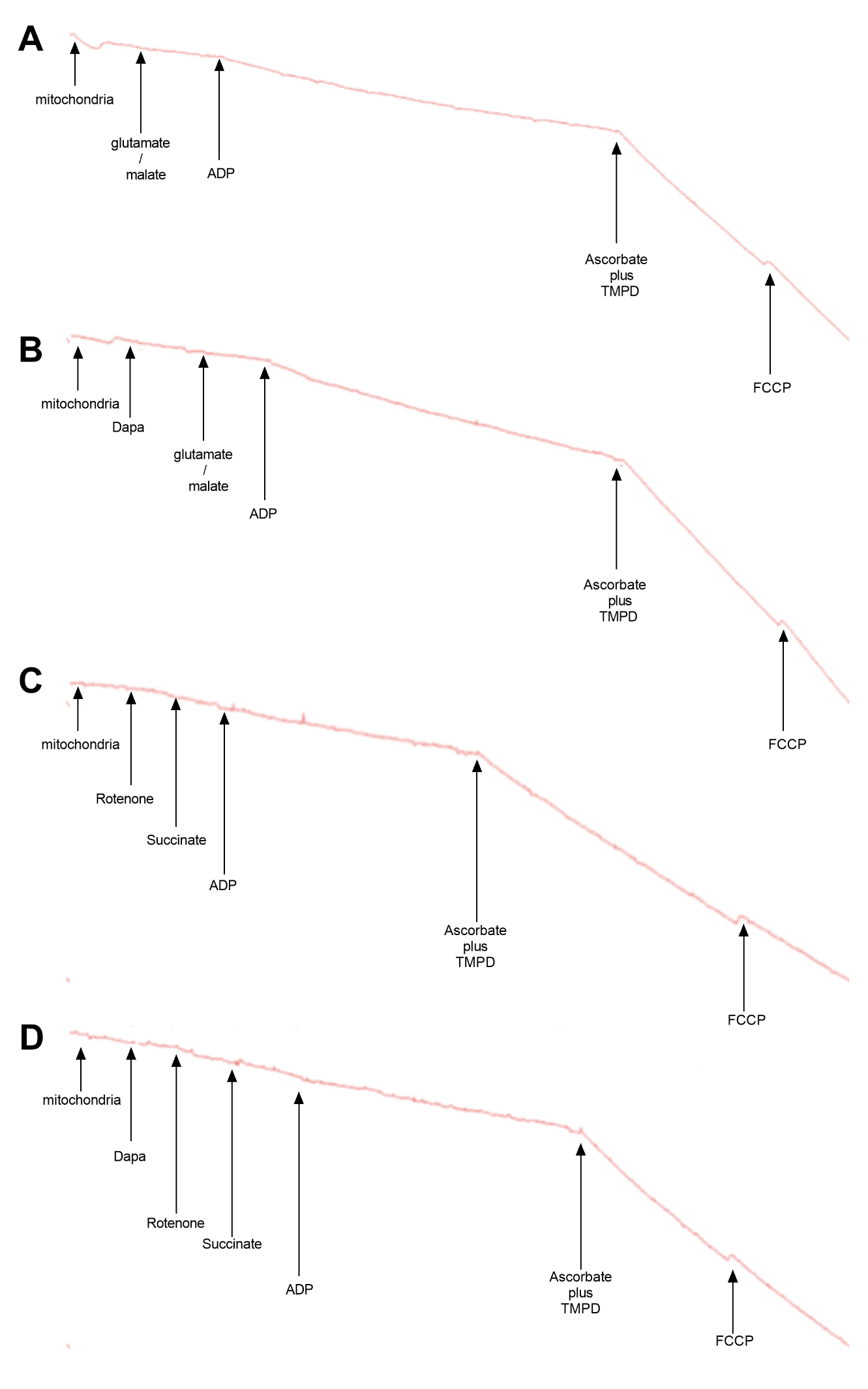


Supplementary figure 1: Representative tracings of oxygen consumption in isolated mitochondria from rat hearts. (A) Oxygen consumption with isolated mitochondria in the presence of: glutamate and malate (5 mM each; state 2, complex I, 1 min), addition of ADP (1 mM; phosphorylating state, 2–5 min), addition of ascorbate (3 µM) and TMPD (300 µM; complex IV electron donors, 1 min), addition of FCCP (30 nM; uncoupler, 1 min). (B) Oxygen consumption with isolated mitochondria in the presence of: Dapagliflozin 10 nM(1 min), addition of glutamate and malate (5 mM each; state 2, complex I, 1 min), addition of ADP (1 mM; phosphorylating state, 2–5 min), addition of ascorbate (3 µM) and TMPD (300 µM; complex IV electron donors, 1 min), addition of FCCP (30 nM; uncoupler, 1 min). (C) Oxygen consumption with isolated mitochondria in the presence of: rotenone (1 µM), and succinate (5 mM)-stimulated respiration (state 2, complex II), ADP (1 mM)-stimulated respiration (state 3, complex II; phosphorylative state), maximal uncoupled respiration induced by FCCP (30 nM), and complex IV respiration stimulated by TMPD (300 µM) and ascorbate (3 µM) in isolated mitochondria from rat hearts. (D) Oxygen consumption with isolated mitochondria in the presence of: Dapagliflozin (10 nM), rotenone (1 µM), and succinate (5 mM)-stimulated respiration (state 2, complex II), ADP (1 mM)-stimulated respiration (state 3, complex II; phosphorylative state), maximal uncoupled respiration induced by FCCP (30 nM), and complex IV respiration stimulated by TMPD (300 µM) and ascorbate (3 µM)
